# Supplementary material for: Validity and usefulness of the student-athletes’ motivation toward sport and academics questionnaire: a Bayesian multilevel approach
Source: PeerJ. 2021 Jul 30;9:e11863. doi: 10.7717/peerj.11863 (PMC8327968; doi:10.7717/peerj.11863)
Supplement: Supplemental Information 1 — Portuguese version of the Student-athletes’ motivation toward sport and academics questionnaire [file peerj-09-11863-s001.docx]

**Questionário acerca da motivação de estudantes-atletas para o esporte e estudo**

| Nas questões seguintes indique seu grau de **CONCORDÂNCIA**, considerando uma escala de 1 a 6, em que **1** é **DISCORDO TOTALMENTE** e **6** **CONCORDO TOTALMENTE**. |
| --- |

| 1 | Estou confiante que este ano posso atingir uma média acadêmica superior a 7. | 1 | 2 | 3 | 4 | 5 | 6 |
| --- | --- | --- | --- | --- | --- | --- | --- |
| 2 | Atingir elevado nível de performance esportiva é um objetivo para mim este ano. | 1 | 2 | 3 | 4 | 5 | 6 |
| 3 | Para mim é importante aprender aquilo que é ensinado no curso. | 1 | 2 | 3 | 4 | 5 | 6 |
| 4 | Estou disposto a dedicar o meu tempo a esforçar-me para obter excelentes notas no meu curso. | 1 | 2 | 3 | 4 | 5 | 6 |
| 5 | Acho mais desafiante enfrentar tarefas complexas estando num ambiente acadêmico. | 1 | 2 | 3 | 4 | 5 | 6 |
| 6 | Para mim os estudos são importantes para atingir conhecimento e competências. | 1 | 2 | 3 | 4 | 5 | 6 |
| 7 | Eu vou estar apto a utilizar o que me foi ensinado no curso em diferentes aspetos da minha vida fora do ambiente escolar. | 1 | 2 | 3 | 4 | 5 | 6 |
| 8 | Eu escolho praticar esporte porque é algo em que quero investir como carreia. | 1 | 2 | 3 | 4 | 5 | 6 |
| 9 | Para mim é importante treinar arduamente de forma a melhorar a minha performance. | 1 | 2 | 3 | 4 | 5 | 6 |
| 10 | Optei ou irei optar por me especializar numa área porque estou interessado(a) nisso como carreira. | 1 | 2 | 3 | 4 | 5 | 6 |
| 11 | Atingir uma média acima de 8 não é um objetivo importante para mim este ano. | 1 | 2 | 3 | 4 | 5 | 6 |
| 12 | Para mim é importante aprender as competências e estratégias que os meus treinadores me ensinam. | 1 | 2 | 3 | 4 | 5 | 6 |
| 13 | É importante para mim ser melhor do que os outros atletas da minha modalidade. | 1 | 2 | 3 | 4 | 5 | 6 |
| 14 | O tempo que eu dispendo a praticar a minha modalidade é satisfatório para mim. | 1 | 2 | 3 | 4 | 5 | 6 |
| 15 | O esforço que faço para ser um atleta excepcional na minha modalidade vale a pena. | 1 | 2 | 3 | 4 | 5 | 6 |
| 16 | A obtenção de um diploma do Ensino Superior é importante para enriquecer o meu conhecimento. | 1 | 2 | 3 | 4 | 5 | 6 |
| 17 | A nível esportivo, acho estimulantes as situações difíceis que requerem performances elevadas e são difícil de realizar. | 1 | 2 | 3 | 4 | 5 | 6 |
| 18 | Durante o tempo todo que competi, obter um diploma não foi um objetivo para mim. | 1 | 2 | 3 | 4 | 5 | 6 |
| 19 | Estou confiante que posso ser um atleta de ponta na minha equipe/modalidade esta temporada. | 1 | 2 | 3 | 4 | 5 | 6 |
| 20 | O meu objetivo esportivo é atingir um nível profissional ou atingir mínimos para os Jogos Olímpicos na minha modalidade. | 1 | 2 | 3 | 4 | 5 | 6 |
| 21 | Acho que as situações que testam as minhas capacidades são desafiantes. | 1 | 2 | 3 | 4 | 5 | 6 |
| 22 | Estou confiante que posso atingir um nível de elite/profissional na minha modalidade. | 1 | 2 | 3 | 4 | 5 | 6 |
| 23 | Estou confiante que posso obter um diploma de conclusão de curso. | 1 | 2 | 3 | 4 | 5 | 6 |
| 24 | Vou conseguir utilizar competências que adquiri no Esporte em outras áreas da minha vida externa ao Esporte. | 1 | 2 | 3 | 4 | 5 | 6 |
| 25 | Atingir resultados esportivos elevados não é um objetivo pessoal para este ano. | 1 | 2 | 3 | 4 | 5 | 6 |
| 26 | Para mim é importante atingir performances elevadas e não cometer erros. | 1 | 2 | 3 | 4 | 5 | 6 |
| 27 | Estou disposto a utilizar o tempo para ser excepcional na minha modalidade. | 1 | 2 | 3 | 4 | 5 | 6 |
| 28 | Maior parte dos conteúdos curriculares do meu curso são interessantes para mim. | 1 | 2 | 3 | 4 | 5 | 6 |
| 29 | É importante obter um diploma universitário porque isso irá ajudar-me a encontrar emprego. | 1 | 2 | 3 | 4 | 5 | 6 |
| 30 | O esforço que faço para obter excelente notas no meu curso não é valioso para mim. | 1 | 2 | 3 | 4 | 5 | 6 |
